# Health and genetic ancestry testing: time to bridge the gap

**DOI:** 10.1186/s12920-016-0240-3

**Published:** 2017-01-09

**Authors:** Andrew Smart, Deborah A. Bolnick, Richard Tutton

**Affiliations:** 1 Department of Sociology, Bath Spa University, Newton Park, Bath, BA2 9BN UK; 2 Department of Anthropology, University of Texas at Austin, 2201 Speedway, Stop C3200, Austin, TX 78712-1723 USA; 3 Department of Sociology, Lancaster University, Bowland North, Bailrigg, LA1 4YN UK

**Keywords:** Direct-to-consumer genetic tests, Genetic ancestry, Disease/Health risk, Regulation, Social implications, Public understanding

## Abstract

**Background:**

It is becoming increasingly difficult to keep information about genetic ancestry separate from information about health, and consumers of genetic ancestry tests are becoming more aware of the potential health risks associated with particular ancestral lineages. Because some of the proposed associations have received little attention from oversight agencies and professional genetic associations, scientific developments are currently outpacing governance regimes for consumer genetic testing.

**Main text:**

We highlight the recent and unremarked upon emergence of biomedical studies linking markers of genetic ancestry to disease risks, and show that this body of scientific research is becoming part of public discourse connecting ancestry and health. For instance, data on genome-wide ancestry informative markers are being used to assess health risks, and we document over 100 biomedical research articles that propose associations between mitochondrial DNA and Y chromosome markers of genetic ancestry and a wide variety of disease risks. Taking as an example an association between coronary heart disease and British men belonging to Y chromosome haplogroup I, we show how this science was translated into mainstream and online media, and how it circulates among consumers of genetic tests for ancestry. We find wide variations in how the science is interpreted, which suggests the potential for confusion or misunderstanding.

**Conclusion:**

We recommend that stakeholders involved in creating and using estimates of genetic ancestry reconsider their policies for communicating with each other and with the public about the health implications of ancestry information.

**Electronic supplementary material:**

The online version of this article (doi:10.1186/s12920-016-0240-3) contains supplementary material, which is available to authorized users.

## Background

While genetic ancestry tests marketed to consumers do not currently claim to provide information about disease risk, it is becoming increasingly difficult to keep information about ancestry separate from information about health. In this article, we consider the recent and unremarked upon growth in genetic tests and biomedical studies linking markers of genetic ancestry to various diseases and medical conditions. These developments are becoming part of public discourse connecting ancestry and health, but because genetic testing companies, oversight agencies, and professional genetic associations have largely treated health and ancestry genetic tests as independent and distinct, little guidance is available to help consumers understand and interpret the reported connections between genetic ancestry and disease risk. Consequently, when such findings circulate in the public realm, consumers learn that there may be health risks tied to their genetic ancestry even though companies do not report those associations. There is therefore potential for confusion or misunderstanding that is problematic for both consumers and the scientific community. We argue that the various stakeholders in genetic ancestry testing need to reconsider what they communicate about the health implications of ancestry information, both to the public and to each other, in order to effectively bridge the gap that currently exists in policies and consumer guidance regarding genetic tests for ancestry and health.

## The gap between genetic tests for health and ancestry

Genetic ancestry tests were first marketed directly to consumers in 2000, for the purpose of reconstructing genealogies and investigating personal genetic heritage. They quickly became the most popular of all consumer genetic testing services, and more than three million individuals have reportedly purchased these tests to date [1, 2]. Over the last 15 years, companies [3], regulators [4] and professional scientific associations [5, 6] have treated ancestry genetic tests differently than medical or health-oriented genetic tests. Tests that make health-related claims or have implications for the prevention, diagnosis, or treatment of disease have been subject to greater scrutiny and oversight, as regulators have sought to ensure that potentially life-changing healthcare decisions are not made on the basis of poor quality information or with a lack of appropriate medical knowledge, advice, and support [7]. Genetic ancestry tests have received less attention from legislators, policy makers and regulatory agencies because they are not marketed explicitly for disease diagnosis, treatment, or prevention, and have thus been seen as more “recreational”, less consequential, and less ethically problematic. This differentiation between ancestry and health genetic testing has seemed appropriate because the two types of tests have had such different applications.

However, recent developments demonstrate that the boundary between ancestry-related and health-related genetic testing is more porous than previously suggested [8], and it is being transgressed in a variety of ways. When genetic testing company 23andMe suspended its ‘health reports’ in 2013, following a warning from the US Food and Drug Administration (FDA), it continued to provide customers with ancestry information and their raw genetic data [9, 10]. With these ancestry testing data, consumers could still obtain an assessment of their health risks using independent online ‘interpretation-only’ services for as little as $5 [11]. More explicit links between genetic ancestry and health are evident in 23andMe’s re-launched Health + Ancestry Service, which was approved by the FDA as a ‘medical device’ and provides both ancestry and health information. This service directly connects ancestry to health in its ‘wellness’ reports for traits like lactose intolerance and in its ‘carrier status’ reports for medical conditions like cystic fibrosis and sickle cell anemia, as both link risk estimates to named racial/ethnic groups. At least one other leading genetic testing company (Ancestry.com) is in discussions with the FDA about expanding its service to include similar carrier status reports [12]. Furthermore, as we show in this article, even when genetic ancestry tests report only an individual’s ancestry, consumers can become aware of possible health risks tied to their genetic ancestry via media coverage of scientific studies and online discussion groups.

This blurring of the line between genetic ancestry and health accentuates the gaps that currently exist in policy and in the available guidance for consumers because little attention has been given to the health implications of genetic ancestry testing. However, as others have noted, it may become common for consumers to share their ancestry test results or ancestry-related estimates of disease risk with their physicians, expecting such information to inform their healthcare decisions and improve their quality of care [13, 14]. It is therefore crucial that we bridge these gaps to ensure that genetic testing information is used appropriately in health-related decisions and clinical care. This is especially important because most physicians lack the expertise needed to interpret and contextualize the results of genetic tests: only 29% of US clinicians surveyed rate their knowledge of genetics as excellent, very good, or good [15], and less than a third of the physicians surveyed in five European countries were confident or very confident in their ability to carry out basic medical genetic tasks [16]. Given these findings, there is a real risk that consumers or their physicians could make problematic and potentially irreversible healthcare decisions based on inaccurate, misleading, or misinterpreted genetic testing results. Genetic ancestry information has been misinterpreted or over-interpreted in the past [17–19], and it has been used in ways that reach far beyond the intended or anticipated scientific applications — for example, in controversial attempts to use genetic ancestry tests to support Native American tribal membership claims [20] and to infer nationality in asylum cases [21].

Thus, it is critical that we recognize and address the increasingly porous boundary between genetic tests for ancestry and health because (1) genetic ancestry tests can provide information that has consequences for health decision-making, (2) test-takers may have unrealistic expectations about the scientific and medical certainties offered by the tests they have purchased, (3) many physicians are not prepared to interpret and apply the genetic test results that their patients may bring into the clinic, and (4) better guidance for consumers is needed to ensure that health-related information from genetic ancestry tests is interpreted and applied in valid ways. This is especially important because, as we show in the next section, scientific and biomedical studies have been drawing ever more connections between ancestry and health, and there is evidence that these connections are beginning to affect consumer interpretations of their genetic ancestry test results.

## Mounting evidence of connections between genetic ancestry and disease risk

Genetic ancestry plays an important role in contemporary biomedical science. Medical genetic studies, for instance, commonly use ancestry inferences derived from autosomal markers (typically single nucleotide polymorphisms or “ancestry informative markers”) to control for population stratification, a practice that underpins the now routine reporting of population-specific or ancestry-specific estimates of disease risks and drug response in genetic epidemiology [13, 22]. Far less attention has been given to the fact that studies using uniparental genetic markers have uncovered connections between ancestry and health. These tests have been a mainstay of the direct-to-consumer ancestry-testing marketplace, but have been widely considered to have little biomedical value [13].

Over the last decade, hundreds of biomedical studies have been published that suggest that certain mitochondrial DNA (mtDNA) and Y chromosome variants (and the haplogroups defined by those variants) are associated with an increased risk of disease and other health complications [23]. These variants and haplogroups have been linked to a diverse array of common diseases and medical conditions, including coronary artery disease, myocardial infarction, ischaemic stroke, heart transplant complications, Leber hereditary optic neuropathy, advanced age-related macular degeneration, hearing loss, osteoarthritis, osteoporosis, multiple sclerosis, Alzheimer’s and Parkinson’s disease, complications from type 2 diabetes (especially retinopathy, neuropathy, nephropathy, and renal failure), several types of cancer (breast, thyroid, pancreatic, esophageal, colorectal, prostate, renal, and lung cancer), and the rate of AIDS progression in HIV patients (an additional table shows examples of associations discussed in the biomedical literature [see Additional file 1]). The exact causes of these associations are not always clear, but it is thought that the associated genetic variants alter the expression of key gene pathways [24] or, in the case of mtDNA, contribute to the development and progression of disease by affecting energy metabolism and important cellular processes, including ATP synthesis, reactive oxygen species (ROS) production, oxygen consumption efficiency, calcium signaling, and apoptosis [25–27].

It is important to note that the quality of studies reporting associations between mtDNA/Y chromosome haplogroups and disease susceptibility is quite variable. Some haplogroup-disease associations are supported by multiple independent studies with rigorous statistical designs, but many others are not. The biomedical literature includes a large number of studies that suffer from small sample sizes, inappropriate controls for population stratification, and/or problematic statistics (for example, *P*-values that have not been corrected for multiple comparisons), so some of the reported haplogroup-disease associations are almost certainly false positives. Other researchers have also drawn attention to these problems [23, 28]. However, regardless of the quality of these studies, many publications present possible associations between mtDNA/Y haplogroups and disease risk, and consumers are becoming aware of these reports as they garner media attention and enter public discourse.

In 2012, for example, a study published in *The Lancet* found that British men belonging to Y chromosome haplogroup I have a 50% higher age-adjusted risk of coronary artery disease (CAD) than other British men, with haplogroup I being the most significant predictor of CAD after HDL cholesterol and lipid-lowering treatment [24]. This finding was widely reported by the media in the UK, US, and Australia, under headlines about heart disease risk being inherited along paternal lines (Tables 1 and 2). This coverage demonstrated the potential for raised consumer expectations about the value of genetic ancestry information to health, and the future possibilities of acting on that information. One article, for example, suggested that: “when a screening test is developed to find those Y chromosome gene clusters and researchers have a better understanding of how they act, it may be possible to protect some [unlucky men] from having heart attacks.” [29] The UK National Health Service added an extensive discussion of ancestral haplogroups and CAD to their patient information website, *NHS Choices*, after the study was published — albeit to argue that this information was not of immediate use for tackling CAD in the UK because, among other reasons, “men are unlikely to know their specific haplogroup, so are unlikely to know whether they may be at increased risk of CAD” [30]. Ancestry testing consumers discussed this *Lancet* study (along with other reports of haplogroup-associated disease susceptibilities) in online forums (in threads entitled, for example, “Medical conditions associated with Y-chromosome haplogroups” and “Do not read if you are a hypochondriac…”), and a Principal Scientist at 23andMe blogged about the study, expressing skepticism about the study’s conclusions and offering an alternative analysis using his company’s data (Table 3). Thus, by following the circulation of this study in public domains, we can see variation in how the study was understood, disagreement over the robustness of its conclusions, and a lack of clarity about the significance of genetic ancestry markers like haplogroups to health.

**Table 1:** Coverage of Charchar et al. [24] in mainstream news media

Outlet	Author	Date	Headline	Section	URL	Quoted
BBC News Online (UK)	Michelle Roberts	09/02/12	Men can inherit a form of heart disease from father via Y chromosome	Health	www.bbc.co.uk/news/health-16931585	Dr Maciej Tomaszewski (University of Leicester); Dr Hélène Wilson (British Heart Foundation)
The Independent (UK)	Jeremy Laurance	09/02/12	Male gene increases risk of hereditary heart disease for one in five	Health	www.independent.co.uk/life-style/health-and-families/ health-news/male-gene-increases-risk-of-hereditary-heart-disease-for-one-in-five-6676537.html	Dr Maciej Tomaszewski (University of Leicester); Dr Virginia M. Miller (Mayo Clinic)
The Daily Telegraph (UK)	Rebecca Smith	09/02/12	One in five men have DNA that puts them at greater risk of a heart attack: research	Health	www.telegraph.co.uk/health/healthnews/9068859/One-in-five-men-have-DNA-that-puts-them-at-greater-risk-of-a-heart-attack-research.html	Dr Hélène Wilson (British Heart Foundation); Dr Maciej Tomaszewski (University of Leicester); Dr Virginia M. Miller (Mayo Clinic)
The Daily Mail (UK)	Sadie Whitelocks	09/02/12	Men can inherit higher risk of heart attack from father - and can pass danger on to their sons	Health	www.dailymail.co.uk/health/article-2098314/Fathers-common-gene-variant-50-cent-higher-risk-heart-disease--pass-sons.html	Dr Hélène Wilson (British Heart Foundation); Dr Maciej Tomaszewski (University of Leicester)
The Daily Mirror (UK)	Lachlan MacKinnon	09/02/12	Close to men’s hearts: Y chromosome link to coronary risk	Technology and science	www.mirror.co.uk/news/technology-science/science/close-to-mens-hearts-y-chromosome-678644	Dr Hélène Wilson (British Heart Foundation); Dr Maciej Tomaszewski (University of Leicester)
Time (USA)	Alexandra Sifferlin	09/02/12	Like Father like Son? Y Chromosome Linked to Heart Disease	Heart Disease	healthland.time.com/2012/02/09/like-father-like-son-y-chromosome-linked-to-heart-disease/	Scientific American Dr Maciej Tomaszewski (University of Leicester)
CBS News (USA)	Brenda Goodman	09/02/12	Some men may inherit a higher risk of heart disease from dad	News	http://www.cbsnews.com/news/some-men-may-inherit-a-higher-risk-of-heart-disease-from-dad/	Lisa Bloomer (University of Leicester); Dr Virginia M. Miller (Mayo Clinic)
The New York Times (USA)	Gina Kolata	08/02/12	Male Genes May Explain Higher Heart Disease Risk	Health	www.nytimes.com/2012/02/09/health/research/heart-disease-risk-may-be-tied-to-y-chromosome.html?_r=0	Dr Virginia M. Miller (Mayo Clinic); Dr Sekar Kathiresan (Massachusetts General Hospital); Dr. Daniel J. Rader (University of Pennsylvania)
Scientific American (USA)	Katherine Haron Courage	08/02/12	Y Chromosome Can Raise Heart Disease Risk by 50%	Blogs	http://blogs.scientificamerican.com/observations/2012/02/08/y-chromosome-can-raise-heart-disease-risk-by-50-percent/	Prof F Charchar (University of Ballarat); Dr Virginia M. Miller (Mayo Clinic)
Forbes (USA)	Larry Husten	09/02/12	The Y Chromosome May Explain Why Men Have Earlier Coronary Disease	Pharma and Healthcare	http://www.forbes.com/sites/larryhusten/2012/02/09/the-y-chromosome-may-explain-why-men-have-earlier-coronary-disease/	The Lancet paper; Dr Virginia M. Miller (Mayo Clinic)
The Age (Australia)	Deb Anderson	27/03/12	Does the Y chromosome influence men’s health?	Education		Prof F Charchar (University of Ballarat)
The Chronicle (Australia)	Tom McIlroy	13/02/12	UB releases heart disease research	News	http://www.thecourier.com.au/story/62162/ub-releases-heart-disease-research/	Prof F Charchar (University of Ballarat); Dr Hélène Wilson (British Heart Foundation)
International Business Times (Australia)	Lawrence Villamar	10/02/12	Heart Disease Risks Linked to Genes in Men: study	Articles		Prof F Charchar (University of Ballarat)
The Times of India (India)	Kounteya Sinha	10/02/12	Sons can inherit heart disease from dad: Study	India	http://timesofindia.indiatimes.com/india/Sons-can-inherit-heart-disease-from-dad-Study/articleshow/11829916.cms	The Lancet paper; Dr Pramod Kumar (Fortis Hospital); Dr Virginia M. Miller (Mayo Clinic)

**Table 2:** Coverage of Charchar et al. [24] in online sources of medical news

Site	Author	Date	Headline	URL	Quoted
NHS Choices		09/02/12	One in five men ‘carries heart risk gene’	www.nhs.uk/news/2012/02February/Pages/y-chromosome-heart-disease-risk.aspx	BBC News
Medical News Today	Jospeh Nordqvist	09/02/12	Male Gene Linked To Coronary Artery Disease Risk	www.medicalnewstoday.com/articles/241441.php	The Lancet paper; Dr Virginia M. Miller (Mayo Clinic)
WebMD	Brenda Goodman	08/02/12	Some Men May Inherit a Higher Risk of Heart Disease From Dad	www.webmd.com/heart-disease/news/20120208/some-men-may-inherit-higher-risk-heart-disease-from-dad	Lisa Bloomer (University of Leicester); Dr Virginia M. Miller (Mayo Clinic)
Medscape	Lisa Nainggolan	10/02/12	Like Father, Like Son: Y-Chromosome Variant May Explain CAD	http://webcache.googleusercontent.com/search?q=cache:RhYvNynICn4J:www.medscape.com/viewarticle/758437+&cd=1&hl=en&ct=clnk&gl=uk	Lisa Bloomer (University of Leicester); Dr Virginia M. Miller (Mayo Clinic); Dr Sekar Kathiresan (Massachusetts General Hospital)
News Medical	AnayaMandal	12/02/12	Y chromosome may be the link that passes heart disease risk from father to son	www.news-medical.net/news/20120 212/Y-chromosome-may-be-the-link-that-passes-heart-disease-risk-from-father-to-son.aspx	The Lancet paper; Dr Sekar Kathiresan (Massachusetts General Hospital); Dr Virginia M. Miller (Mayo Clinic)
The Naked Scientist	Kat Arney	12/02/12	Y Chromosome yields heart disease clues	www.thenakedscientists.com/HTML/news/news/2485/	
MyDr		09/02/12	Heart disease risk passed from father to son		Dr Virginia M. Miller (Mayo Clinic)
Cure Talk	Priya Menon	undated	Coronary Artery Disease Linked To Y Chromosome: Study By Fadi J. Charchar	trialx.com/curetalk/2012/03/coronary-artery-disease-linked-to-y-chromosome-study-by-fadi-j-charchar/	
Men’s Health		09/02/12	Y chromosome may increase risk of coronary artery disease	www.drharryfisch.com/y-chromosome-may-increase-risk-of-coronary-artery-disease/	
drholdright.co.uk		02/12	The Y male sex chromosome and risk of heart disease	http://www.drholdright.co.uk/dynamicpage.php?pg=news&pageid=OTk=

**Table 3:** Coverage linking haplogroups and health online in blogs and forums

Site	Author	Date	Headline	Type	URL	Includes Charchar et al. (2012) [24]
23andMe	Dave Hinds	04/04/12	Second Opinion: Haplogroup I Likely Not Linked to Heart Disease	blog	blog.23andme.com/news/second-opinion-haplogroup-i-likely-not-linked-to-heart-disease/	Yes
Improving Population Health	David A. Kindig	07/10/12	Are Male Genetic Health Differences Disparities or Inequities	blog	www.improvingpopulationhealth.org/blog/2012/07/are-male-genetic-health-differences-disparities-or-inequities.html	Yes
Dienekes Anthropology Blog		09/02/12	Y-chromosomes and coronary artery disease in Britain	blog	dienekes.blogspot.co.uk/2012/02/y-chromosomes-and-coronary-artery.html	Yes
Fight Aging		05/11/13	Those Lucky Haplogroup H Bearers	blog	www.fightaging.org/archives/2013/11/those-lucky-haplogroup-h-bearers.php	No
Mathilda’s Anthropolgy Blog		03/11/08	Mitochondrial DNA and survival after sepsis	blog	mathildasanthropologyblog.wordpress.com/2008/11/03/mitochondrial-dna-and-survival-after-sepsis/	No
Eupedia		28/01/09 – 23/09/13	Medical conditions associated with Y-chromosome haplogroups	forum	www.eupedia.com/forum/threads/25199-Medical-conditions-associated-with-Y-chromosome-haplogroups	Yes
familytreeDNA		09/02/12 – 10/02/12	Do not read if you are a hypochondriac…	forum	forums.familytreedna.com/showthread.php?t = 30303&highlight = haplogroup + health + risk	Yes
Rootschat		22/05/13 – 01/06/13	Utter Confusion: please help me choose DNA test	forum	www.rootschat.com/forum/index.php?topic=647807.54	Yes (via ref to Hinds blog)
Eupedia		26/07/07 – 26/04/14	Medical conditions and risk factors associated with mtDNA haplogroups	forum	www.eupedia.com/forum/threads/24801-Medical-conditions-and-risk-factors-associated-with-mtDNA-haplogroups	No
Zetaboards		12/01/13 – 12/01/13	Examples of diseases associated with a haplogroup	forum	s1.zetaboards.com/anthroscape/topic/5045507/1/	No
WorldFamilies		14/06/11 – 20/03/12	At last some FGS-s for R0a2 from Tuscany and The Marche	forum	www.worldfamilies.net/forum/index.php?topic=9937.0	No

While similar observations can be made about studies of autosomal or genome-wide markers and disease risk, we have focused here on uniparental genetic markers because mtDNA and Y chromosome tests are the two types of genetic ancestry tests that companies, policy-makers, regulators, and professional scientific associations have invariably treated as less relevant to health. However, as we have shown, there is public interest in the extensive biomedical literature investigating the associations between mtDNA/Y chromosome haplogroups and disease, and some consumers of genetic ancestry tests are already trying to understand how their ancestry is relevant to their health and disease prognosis in light of these research findings. Other consumers are also likely to encounter the results of these biomedical studies in the popular press, in online forums, in literature from their healthcare provider, or in well-known medical journals, and they too may grapple with the possible health implications of their genetic ancestry test results. Therefore, even when genetic ancestry tests report only an individual’s ancestral lineage or uniparental haplogroup, consumers can become aware of the possible relationship with genetic health risks because scientists have linked haplogroup ancestry to health outcomes.

## Conclusions

Like the repurposing of genome-wide ancestry test data to assess health risks, and like the use of autosomal genetic markers in genetic epidemiology to identify population-specific disease risks and drug response, the reported associations between uniparentally-inherited haplogroups and various diseases represent another blurring of the line between genetic information on health and ancestry. Developments in scientific knowledge and commercial practice appear to be outpacing the current oversight and governance regimes that largely treat genetic tests for ancestry and health as separate and distinct.

We therefore suggest that it is time for the various stakeholders in genetic ancestry testing to reconsider what they communicate about the health implications of ancestry information, both to the public and to each other. This will require considering some difficult questions. For example, can and should consumer genetic testing companies take responsibility for the ways in which test-takers connect ancestry test results with other publicly available information? Can and should genetic testing companies, alongside scientific associations and consumer advocates, provide guidance to consumers about the accuracy and reliability of the various postulated associations between haplogroups (or other genetic markers) and health risks? How can those who communicate about the relevant science (scientific researchers, journal editors, science journalists, genetic testing companies, consumer advocates, policy advisors, etc.) achieve maximum clarity regarding the potential health implications of genetic ancestry (or the lack of them), and make effective use of the published critiques of the putative links between uniparental haplogroups and health risks [23, 28]? Should there be any changes to policies governing the regulation or oversight of consumer genetic testing, or additions to guidelines being developed by professional associations like the American Society of Human Genetics (ASHG)?

To help address the issues raised here, we make five recommendations for stakeholders in consumer genetic testing to consider:

1. The ASHG or another respected professional genetics society should organize a roundtable to bring the various stakeholders together to produce authoritative guidance that will inform and benefit consumers, and help create a set of standards for the industry to follow. This guidance should make clear what we can and cannot know from genetic ancestry testing, and provide guidance regarding the accuracy and reliability of associations between genetic markers and health risks. Industry representatives, consumer advocates, policy and legal advisors, biomedical researchers, social scientists, and regulators should all be involved in drafting this guidance.
2. Consumer advocates, scientific organizations, companies, science journalists, and government agencies should play a role in making this information available to consumers.
3. Genetic ancestry testing companies should report only associations between genetic markers and diseases/medical conditions that have been scientifically validated. They should also provide information about the limitations of their tests (as 23andMe now does as part of their ‘carrier status’ and ‘wellness’ reports; [31]) and include information about how to interpret the connections between ancestry and health among their FAQs.
4. Medical schools and continuing medical education (CME) programs should discuss the potential health implications of genetic ancestry information so that physicians can help their patients to interpret and contextualize their genetic testing results.
5. Policy-makers and government agencies may wish to reconsider current oversight regimes for direct-to-consumer genetic testing in light of the increasingly porous boundaries between tests for health and ancestry.

Our recommendations are aimed at encouraging novel and timely interventions into ongoing debates about direct-to-consumer genetic tests, especially since the US FDA, European Commission, ASHG, and high-profile ancestry testing companies are all considering scientific, ethical, and regulatory issues regarding health-related genetic testing. Now is the time to start bridging the gap in our current approaches to health and ancestry genetic testing.

## Additional file

Additional file 1: Examples of associations between mitochondrial DNA (mtDNA) or Y chromosome variants and diseases/medical traits discussed in the biomedical literature. Description of data: an extensive list of published associations between mitochondrial DNA (mtDNA) or Y chromosome variants and diseases/medical traits. Organised by disease/medical traits, and including: mtDNA or Y Chromosome Variant; Proposed Association (or Lack of Association); Study Location; and Reference. (DOCX 58 kb)

## Abbreviations

ASHG: American Society of Human Genetics
CAD: Coronary artery disease
CME: Continuing medical education
FDA: Food and Drug Administration
mtDNA: Mitochondrial DNA
PGS: Personal genome service
ROS: Reactive oxygen species
