# Supplementary material for: Health and genetic ancestry testing: time to bridge the gap
Source: BMC Med Genomics. 2017 Jan 9;10:3. doi: 10.1186/s12920-016-0240-3 (PMC5223458; doi:10.1186/s12920-016-0240-3)
Supplement: Additional file 1: — Examples of associations between mitochondrial DNA (mtDNA) or Y chromosome variants and diseases/medical traits discussed in the biomedical literature. Description of data: an extensive list of published associations between mitochondrial DNA (mtDNA) or Y chromosome variants and diseases/medical traits. Organised by disease/medical traits, and including: mtDNA or Y Chromosome Variant; Proposed Association (or Lack of Association); Study Location; and Reference. (DOCX 58 kb) [file 12920_2016_240_MOESM1_ESM.docx]

**Examples of associations between mitochondrial DNA (mtDNA) or Y chromosome variants and diseases/medical traits discussed in the biomedical literature.**

*Note: Published associations were included regardless of the quality of statistical analysis or the robustness of the reported association. Some of the associations listed here are likely false positives. This is not a comprehensive listing of all relevant studies and does not include all reported associations.*

| ***mtDNA or Y Chromosome Variant*** | ***Proposed Association (or Lack of Association)*** | | ***Study Location*** | | ***Reference*** | |
| --- | --- | --- | --- | --- | --- | --- |
| **Longevity** |  | |  | |  | |
| mtDNA haplogroup D4 | longevity | | China | | X. Y. Cai, *et al.*, Association of mitochondrial DNA haplogroups with exceptional longevity in a Chinese population. PloS ONE **4**, e6423 (2009), 10.1371/journal.pone.0006423. | |
| mtDNA haplogroups M9, N9, B4a | reduced longevity | | China | | X. Y. Cai, *et al.*, Association of mitochondrial DNA haplogroups with exceptional longevity in a Chinese population. PloS ONE **4**, e6423 (2009), 10.1371/journal.pone.0006423. | |
| mtDNA 150T | longevity | | Costa Rica | | L. Castri *et al.*, Mitochondrial polymorphisms associated with differential longevity do not impact lifetime-reproductive success. *Am J Hum Biol* **23**, 225-227 (2011). | |
| mtDNA 5178A in haplogroup D | reduced longevity | | Costa Rica | | L. Castri *et al.*, Mitochondrial polymorphisms associated with differential longevity do not impact lifetime-reproductive success. *Am J Hum Biol* **23**, 225-227 (2011). | |
| mtDNA haplogroups D4a, D5, D4b2b | longevity | | Japan, Korea | | Y. Nishigaki, N. Fuku, M. Tanaka, Mitochondrial haplogroups associated with lifestyle-related diseases and longevity in the Japanese population. *Geriatr Gerontol Int* **10 Suppl 1**, S221-235 (2010). | |
| mtDNA haplogroup F | longevity | | China | | J. Feng *et al.*, Association of mtDNA haplogroup F with healthy longevity in the female Chuang population, China. *Exp Gerontol* **46**, 987-993 (2011). | |
| mtDNA 150T | no association with longevity | | China | | H. Pan *et al.*, Absence of association between mitochondrial DNA C150T polymorphism and longevity in a Han Chinese population. *Exp Gerontol* **46**, 511-515 (2011). | |
| ***mtDNA or Y Chromosome Variant*** | ***Proposed Association (or Lack of Association)*** | | ***Study Location*** | | ***Reference*** | |
| **Longevity (cont.)** |  | |  | |  | |
| mtDNA haplogroups | no association with longevity | | Spain | | T. Pinos *et al.*, Are mitochondrial haplogroups associated with extreme longevity? A study on a Spanish cohort. *Age (Dordr)* **34**, 227-233 (2012). | |
| mtDNA haplogroup X | successful aging | | USA | | M. D. Courtenay *et al.*, Mitochondrial haplogroup X is associated with successful aging in the Amish. *Hum Genet* **131**, 201-208 (2012). | |
| mtDNA haplogroup J | less successful aging | | USA | | M. D. Courtenay *et al.*, Mitochondrial haplogroup X is associated with successful aging in the Amish. *Hum Genet* **131**, 201-208 (2012). | |
| mtDNA 150T, 73G | longevity | | Turkey | | O. Guney *et al.*, Mitochondrial DNA polymorphisms associated with longevity in the Turkish population. *Mitochondrion* **17**, 7-13 (2014). | |
| mtDNA haplogroup D4b2 and 1382C | longevity | | Japan | | N. Fuku *et al.*, The mitochondrial-derived peptide MOTS-c: a player in exceptional longevity? *Aging Cell* **14**, 921-923 (2015). | |
| mtDNA haplogroups | no association with longevity | | China | | Y.-H. He *et al.*, Mitochondrial DNA plays an equal role in influencing female and male longevity in centenarians. *Exp Geront* **83**, 94-96 (2016). | |
| mtDNA haplogroup M9 | reduced longevity | | China | | L. Li *et al.*, Mitochondrial genomes and exceptional longevity in a Chinese population: the Rugao longevity study. *Age* **37**, 14 (2015). | |
| mtDNA haplogroups A4h, R11a1a1a | longevity | | China | | L. Li *et al.*, Mitochondrial genomes and exceptional longevity in a Chinese population: the Rugao longevity study. *Age* **37**, 14 (2015). | |
| mtDNA haplogroup C | reduced longevity | | Costa Rica | | L. Castri *et al.*, A mitochondrial haplogroup is associated with decreased longevity in a historic New World population. *Hum Biol* **86**, 251-259 (2014). | |
| **Cancer** |  | |  | |  | |
| mtDNA 239C, 263G, 16207T, haplogroup I | breast cancer | | Poland | | A. M. Czarnecka *et al*, Mitochondrial genotype and breast cancer predisposition. *Oncol Rep* **24**, 1521-1534 (2010). | |
| mtDNA 73G, 150T, 16183C, 16189C, 16223T, 16362C | reduced risk of breast cancer | | Poland | | A. M. Czarnecka *et al*, Mitochondrial genotype and breast cancer predisposition. *Oncol Rep* **24**, 1521-1534 (2010). | |
| ***mtDNA or Y Chromosome Variant*** | ***Proposed Association (or Lack of Association)*** | | ***Study Location*** | | ***Reference*** | |
| **Cancer (cont.)** |  | |  | |  | |
| linked mtDNA 16183C, 16189C, 16192T, 16270T, 195T | malignant melanoma | | Middle Europe | | S. Ebner *et al.*, Mitochondrial haplogroups, control region polymorphisms and malignant melanoma: a study in middle European Caucasians. *PLoS One* **6**, e27192 (2011), 10.1371/journal.pone.0027192. | |
| mtDNA haplogroups M, D5 | breast cancer | | China | | H. Fang *et al.*, Cancer type-specific modulation of mitochondrial haplogroups in breast, colorectal and thyroid cancer. *BMC Cancer* **10**, 421 (2010). | |
| mtDNA haplogroup D4a | thyroid cancer | | China | | H. Fang *et al.*, Cancer type-specific modulation of mitochondrial haplogroups in breast, colorectal and thyroid cancer. *BMC Cancer* **10**, 421 (2010). | |
| mtDNA haplogroup Uk | vulvar squamous cell carcinoma | | Poland | | A. Klemba *et al*., Mitochondrial genotype in vulvar carcinoma - cuckoo in the nest. *J Biomed Sci* **17**, 73 (2010). | |
| mtDNA haplogroup H | reduced risk of vulvar squamous cell carcinoma | | Poland | | A. Klemba *et al*., Mitochondrial genotype in vulvar carcinoma - cuckoo in the nest. *J Biomed Sci* **17**, 73 (2010). | |
| various rare mtDNA variants | pancreatic cancer | | USA | | E. T. Lam *et al.*, Mitochondrial DNA sequence variation and risk of pancreatic cancer. *Cancer Res* **72**, 686-695 (2012). | |
| mtDNA haplogroup D (especially D4a, D5) | esophageal cancer | | China | | X. Y. Li *et al.*, Association of mitochondrial haplogroup D and risk of esophageal cancer in Taihang Mountain and Chaoshan areas in China. *Mitochondrion* **11**, 27-32 (2011). | |
| mtDNA haplogroup U | prostate cancer | | USA | | L. M. Booker *et al*, North American white mitochondrial haplogroups in prostate and renal cancer. *J Urol* **175**, 468-472 (2006). | |
| mtDNA haplogroup U | no association with prostate cancer | | Middle Europe | | E. E. Mueller *et al.*, Mitochondrial haplogroups and control region polymorphisms are not associated with prostate cancer in Middle European Caucasians. *PLoS One* **4**, e6370 (2009), 10.1371/journal.pone.0006370. | |
| mtDNA haplogroup M | breast cancer | | China | | L. Shen *et al.*, Evaluating mitochondrial DNA in cancer occurrence and development. *Ann N Y Acad Sci* **1201**, 26-33 (2010). | |
| ***mtDNA or Y Chromosome Variant*** | ***Proposed Association (or Lack of Association)*** | | ***Study Location*** | | ***Reference*** | |
| **Cancer (cont.)** |  | |  | |  | |
| mtDNA haplogroup M5 | breast cancer | | India | | N. R. Tipirisetti *et al.*, Mitochondrial genome variations in advanced stage breast cancer: a case-control study. *Mitochondrion* **13**, 372-378 (2013). | |
| mtDNA 752A, 1440A, 4770A | colorectal cancer | | Scotland | | E. Theodoratou *et al.*, Association between common mtDNA variants and all-cause or colorectal cancer mortality. *Carcinogenesis* **31**, 296-301 (2010). | |
| mtDNA haplogroups D, F | reduced risk of lung cancer | | China | | S. Zheng *et al.*, Association of mitochondrial DNA variations with lung cancer risk in a Han Chinese population from southwestern China. *PLoS One* **7**, e31322 (2012), 10.1371/journal.pone.0031322. | |
| mtDNA haplogroups G, M7 | lung cancer | | China | | S. Zheng *et al.*, Association of mitochondrial DNA variations with lung cancer risk in a Han Chinese population from southwestern China. *PLoS One* **7**, e31322 (2012), 10.1371/journal.pone.0031322. | |
| mtDNA haplogroup JT | myelodysplastic syndromes | | USA | | J. N. Poynter *et al*., Association between mitochondrial DNA haplogroup and myelodysplastic syndromes. *Genes Chromosomes Cancer* **55**, 9 (2016), 10.1002/gcc.22370. | |
| mtDNA haplogroups | no association with prostate cancer | | Colombia | | D. Cano *et al*., Mitochondrial DNA haplogroups and susceptibility to prostate cancer in a colombian population. *ISRN Oncol*. 530675 (2014), 10.1155/2014/530675. | |
| mtDNA | no association with prostate cancer | | USA | | E. E. Giorgi *et al*., No Association between the Mitochondrial Genome and Prostate Cancer Risk: The Multiethnic Cohort. Cancer Epidemiol Biomarkers Prev. **25**, 6 (2016), 10.1158/1055-9965. | |
| mtDNA haplogroup T | colorectal cancer | | USA | | Y. Li *et al.,* Association of Genes, Pathways, and Haplogroups of the Mitochondrial Genome with the Risk of Colorectal Cancer: The Multiethnic Cohort. PLoS One **10**, e0136796 (2015), 10.1371/journal.pone.0136796. | |
| mtDNA haplogroup H | breast cancer | | Uruguay | | C. Bonilla *et al*, Breast cancer risk and genetic ancestry: a case-control study in Uruguay. *BMC Womens Health*. **15**, 11 (2015) 10.1186/s12905-015-0171-8. | |
| ***mtDNA or Y Chromosome Variant*** | ***Proposed Association (or Lack of Association)*** | | ***Study Location*** | | ***Reference*** | |
| **Cancer (cont.)** |  | |  | |  | |
| mtDNA haplogroups R9, F1 | nasopharyngeal carcinoma | | China | | S. P. Hu, J. P. Du, D. R. Li, Y. G. Yao, Mitochondrial DNA haplogroup confers genetic susceptibility to nasopharyngeal carcinoma in Chaoshanese from Guangdong, China. PLoS One **9**, e87795 (2014), 10.1371/journal.pone.0087795. | |
| **Coronary Conditions** |  | |  | |  | |
| mtDNA haplogroups N9b, M7c | reduced risk of myocardial infarction | | Japan, Korea | | Y. Nishigaki, N. Fuku, M. Tanaka, Mitochondrial haplogroups associated with lifestyle-related diseases and longevity in the Japanese population. *Geriatr Gerontol Int* **10 Suppl 1**, S221-235 (2010). | |
| mtDNA haplogroup G1 | myocardial infarction | | Japan, Korea | | Y. Nishigaki, N. Fuku, M. Tanaka, Mitochondrial haplogroups associated with lifestyle-related diseases and longevity in the Japanese population. *Geriatr Gerontol Int* **10 Suppl 1**, S221-235 (2010). | |
| mtDNA 16189C | coronary artery disease & myocardial infarction | | Saudi Arabia | | K. K. Abu-Amero *et a.l*, The mitochondrial DNA variant 16189T>C Is associated with coronary artery disease and myocardial infarction in Saudi Arabs. *Genet Test Mol Bioma* **14**, 43-47 (2010). | |
| mtDNA haplogroup H | reduced risk of left ventricular hypertrophy | | Russia | | S. V. Buikin, M. V. Golubenko, V. P. Puzyrev, Genes for mitochondria in arterial hypertension and left ventricular hypertrophy. *Mol Biol* **44**, 23-27 (2010). | |
| mtDNA haplogroup T | left ventricular hypertrophy | | Russia | | S. V. Buikin, M. V. Golubenko, V. P. Puzyrev, Genes for mitochondria in arterial hypertension and left ventricular hypertrophy. *Mol Biol* **44**, 23-27 (2010). | |
| Y chromosome haplogroup I | coronary artery disease | | UK | | F. J. Charchar *et al.*, Inheritance of coronary artery disease in men: an analysis of the role of the Y chromosome. *Lancet* **379**, 915-922 (2012). | |
| mtDNA haplogroup K | reduced risk of transient ischaemic attack, & ischaemic stroke | | UK | | P. F. Chinnery *et al.*, Mitochondrial DNA haplogroups and risk of transient ischaemic attack and ischaemic stroke: a genetic association study. *Lancet Neurol* **9**, 498-503 (2010). | |
| ***mtDNA or Y Chromosome Variant*** | ***Proposed Association (or Lack of Association)*** | | ***Study Location*** | | ***Reference*** | |
| **Coronary Conditions (cont.)** | |  | |  | |  |
| mtDNA haplogroup H | end-stage heart failure | | Europe | | M. E. Gallardo *et al.*, Mitochondrial haplogroups associated with end-stage heart failure and coronary allograft vasculopathy in heart transplant patients. *Eur Heart J* **33**, 346-353 (2012). | |
| mtDNA haplogroup Uk | cardiac allograft vasculopathy | | Europe | | M. E. Gallardo *et al.*, Mitochondrial haplogroups associated with end-stage heart failure and coronary allograft vasculopathy in heart transplant patients. *Eur Heart J* **33**, 346-353 (2012). | |
| mtDNA haplogroup W | coronary artery disease | | Lebanon | | M. Haber *et al.*, mtDNA lineages reveal coronary artery disease-associated structures in the Lebanese population. *Ann Hum Genet* **76**, 1-8 (2012). | |
| mtDNA haplogroup T | coronary artery disease | | Austria | | B. Kofler *et al.*, Mitochondrial DNA haplogroup T is associated with coronary artery disease and diabetic retinopathy: a case control study. *BMC Med Genet* **10**, 35 (2009), 10.1186/1471-2350-10-35. | |
| mtDNA 16189C | coronary artery disease | | Austria | | E. E. Mueller *et al.*, The mitochondrial T16189C polymorphism is associated with coronary artery disease in Middle European populations. *PLoS One* **6**, e16455 (2011), 10.1371/journal.pone.0016455. | |
| mtDNA haplogroup H | early-onset myocardial infarction | | Spain | | M. Palacin *et al.*, Mitochondrial DNA and TFAM gene variation in early-onset myocardial infarction: evidence for an association to haplogroup H. *Mitochondrion* **11**, 176-181 (2011). | |
| mtDNA haplogroups A, M7a | coronary atherosclerosis | | Japan | | M. Sawabe *et al.*, Mitochondrial haplogroups A and M7a confer a genetic risk for coronary atherosclerosis in the Japanese elderly: an autopsy study of 1,536 patients. *J Atheroscler Thromb* **18**, 166-175 (2011). | |
| Y chromosome haplogroup K | atherosclerotic plaque occurrence | | Cyprus | | K. Voskarides, D. Hadjipanagi, L. Papazachariou, M. Griffin, A. G. Panayiotou, Evidence for contribution of the Y chromosome in atherosclerotic plaque occurrence in men. *Genet Test Mol Biomarkers* **18**, 552-556 (2014). | |
| ***mtDNA or Y Chromosome Variant*** | ***Proposed Association (or Lack of Association)*** | | ***Study Location*** | | ***Reference*** | |
| **Coronary Conditions (cont.)** | |  | |  | |  |
| Y chromosome haplogroup YAP | reduced risk of atherosclerotic plaque | | Cyprus | | K. Voskarides, D. Hadjipanagi, L. Papazachariou, M. Griffin, A. G. Panayiotou, Evidence for contribution of the Y chromosome in atherosclerotic plaque occurrence in men. *Genet Test Mol Biomarkers* **18**, 552-556 (2014). | |
| Y chromosome haplogroups | no association with cardiovascular risk | | Poland | | G. Kostrzewa, G. Broda, M. Konarzewska, P. Krajewki, R. Płoski. Genetic polymorphism of human Y chromosome and risk factors for cardiovascular diseases: a study in WOBASZ cohort. *PLoS ONE* **8**, e68155 (2013), doi:10.1371/journal.pone.0068155. | |
| mtDNA haplogroup H | hypertrophic cardiomyopathy | | Denmark | | C. M. Hagen *et al.*, Mitochondrial haplogroups modify the risk of developing hypertrophic cardiomyopathy in a Danish population. *PLoS One* **8**, e71904 (2013), 10.1371/journal.pone.0071904. | |
| Y chromosome haplogroups | no association with recurrent venous thrombosis | | Holland | | H. G. de Haan *et al*., Male-specific risk of first and recurrent venous thrombosis: a phylogenetic analysis of the Y chromosome. *J Thromb Haemost*. (2016), doi: 10.1111/jth.13437. | |
| mtDNA haplogroup D4b | reduced risk of ischemic stroke | | China | | D. Yang *et al*., Mitochondrial DNA haplogroup D4b is a protective factor for ischemic stroke in Chinese Han population. *Mol Genet Genomics.* **289**, 6, 1241-6 (2014). | |
| mtDNA haplogroup N9 | facilitates neurological recovery after ischemic stroke | | China | | B. Cai *et al*., Mitochondrial DNA haplogroups and short-term neurological outcomes of ischemic stroke. *Sci Rep* **20**, 5, 9864 (2015), 10.1038/srep09864. | |
| mtDNA haplogroup H | subclinical carotid atherosclerosis | | Russia | | A. Zhelankin *et al.,* 1A.06: Mitochondrial DNA haplogroup H is associated with subclinical carotid atherosclerosis in Russian population. *J Hypertens* **33** suppl 1:e2 (2015), 10.1097/01.hjh.0000467356.04711.5 | |
| mtDNA haplogroups J, Uk | reduced risk of hypertrophic cardiomyopathy | | Denmark | | C. M. Hagen *et al.*, Mitochondrial haplogroups modify the risk of developing hypertrophic cardiomyopathy in a Danish population. *PLoS One* **8**, e71904 (2013), 10.1371/journal.pone.0071904. | |
| ***mtDNA or Y Chromosome Variant*** | ***Proposed Association (or Lack of Association)*** | | ***Study Location*** | | ***Reference*** | |
| **Type 2 Diabetes** |  | |  | |  | |
| mtDNA haplogroups F, D4b | type 2 diabetes | | Japan, Korea | | Y. Nishigaki, N. Fuku, M. Tanaka, Mitochondrial haplogroups associated with lifestyle-related diseases and longevity in the Japanese population. *Geriatr Gerontol Int* **10 Suppl 1**, S221-235 (2010). | |
| mtDNA haplogroups H, H3, U3, V | complications from diabetes | | Italy | | A. Achilli *et al.*, Mitochondrial DNA backgrounds might modulate diabetes complications rather than T2DM as a whole. *PLoS One* **6**, e21029 (2011), 10.1371/journal.pone.0021029. | |
| mtDNA haplogroup N9a | reduced risk of type 2 diabetes | | Japan, Korea | | Y. Nishigaki, N. Fuku, M. Tanaka, Mitochondrial haplogroups associated with lifestyle-related diseases and longevity in the Japanese population. *Geriatr Gerontol Int* **10 Suppl 1**, S221-235 (2010). | |
| mtDNA haplogroup J1 | type 2 diabetes | | Europe, North Africa | | J. Feder *et al.*, Parental diabetes status reveals association of mitochondrial DNA haplogroup J1 with type 2 diabetes. *BMC Med Genet* **10**, 60 (2009), 10.1186/1471-2350-10-60. | |
| mtDNA 16189C | no association with type 2 diabetes | | China | | L. Zhong *et al.*, Reappraising the relationship between mitochondrial DNA variant m.16189t > c and type 2 diabetes mellitus in East Asian populations. *Curr Mol Med* **14**, 1273-1278 (2014). | |
| mtDNA 16390A | type 2 diabetes | | Tunisia | | S. Hsousa, *et al.*, Association study of mitochondrial DNA polymorphisms with type 2 diabetes in Tunisian population. *Mitochondr DNA* **26**, 367-372 (2015). | |
| mtDNA haplogroups M8a, N9a | type 2 diabetes | | China | | Q. Niu *et al.*, Effects of mitochondrial haplogroup N9a on type 2 diabetes mellitus and its associated complications. *Exp Ther Med* **10**, 1918-1924 (2015). | |
| mtDNA 3243G, 16189C | type 2 diabetes | | Asia | | S. H. Kwak, K. S. Park, Role of mitochondrial DNA variation in the pathogenesis of diabetes mellitus. *Front Biosci (Landmark Ed.)* **21**, 1151-1167 (2016). | |
| mtDNA haplogroups and mutations | no association with type 2 diabetes | | Denmark | | S. Li *et al.*, Variation and association to diabetes in 2000 full mtDNA sequences mined from an exome study in a Danish population. *Eur J Hum Genet* **22**, 1040-1045 (2014). | |
| ***mtDNA or Y Chromosome Variant*** | ***Proposed Association (or Lack of Association)*** | | ***Study Location*** | | ***Reference*** | |
| **Hearing Loss** |  | |  | |  | |
| mtDNA haplogroup L1 | noise-induced hearing loss | | Brazil | | R. S. Abreu-Silva *et al.*, The search of a genetic basis for noise-induced hearing loss (NIHL). *Ann Hum Biol* **38**, 210-218 (2011). | |
| mtDNA 1555G | aminoglycoside-induced, non-syndromic hearing loss | | China | | Y. Bai *et al.*, A six-generation Chinese family in haplogroup B4C1C exhibits high penetrance of 1555A > G-induced hearing Loss. *BMC Med Genet* **11**, 129 (2010), 10.1186/1471-2350-11-129. | |
| mtDNA 1555G | non-syndromic sensorineural hearing loss | | Morocco | | H. Nahili *et al.*, Prevalence of the mitochondrial A 1555G mutation in Moroccan patients with non-syndromic hearing loss. *Int J Pediatr Otorhinolaryngol* **74**, 1071-1074 (2010). | |
| mtDNA 1555G | aminoglycoside-induced, non-syndromic hearing loss | | China | | J. Lu *et al.*, Mitochondrial haplotypes may modulate the phenotypic manifestation of the deafness-associated 12S rRNA 1555A>G mutation. *Mitochondrion* **10**, 69-81 (2010). | |
| mtDNA 1555G, haplogroup B | higher risk, penetrance, & expressivity of hearing loss | | China | | J. Lu *et al.*, Mitochondrial haplotypes may modulate the phenotypic manifestation of the deafness-associated 12S rRNA 1555A>G mutation. *Mitochondrion* **10**, 69-81 (2010). | |
| mtDNA 1555G, 961G | non-syndromic sensorineural hearing loss | | Italy | | V. Guaran *et al.*, Association between idiopathic hearing loss and mitochondrial DNA mutations: a study on 169 hearing-impaired subjects. *Int J Mol Med* **32**, 785-794 (2013). | |
| mtDNA 1555G, 3243G, 3595G, 6204G | non-syndromic hearing loss | | Japan | | T. Yano *et al.*, Frequency of mitochondrial mutations in non-syndromic hearing loss as well as possibly responsible variants found by whole mitochondrial genome screening. *J Hum Genet* **59**, 100-106 (2014). | |
| mtDNA 1222G | non-syndromic sensorineural hearing loss | | China | | Q. Wei *et al.*, Genetic mutations of GJB2 and mitochondrial 12S rRNA in nonsyndromic hearing loss in Jiangsu Province of China. *J Transl Med* **11**, 163 (2013). | |
| mtDNA 1555G, haplogroup B | aminoglycoside-induced, non-syndromic hearing loss | | China | | Z. Ying *et al.*, Mitochondrial haplogroup B increases the risk for hearing loss among the Eastern Asian pedigrees carrying 12S rRNA 1555A>G mutation. *Protein Cell* **6**, 844-848 (2015). | |
| ***mtDNA or Y Chromosome Variant*** | ***Proposed Association (or Lack of Association)*** | | ***Study Location*** | | ***Reference*** | |
| **Hearing Loss (cont.)** |  | |  | |  | |
| mtDNA haplogroups D4a, M22, H2 | aminoglycoside-induced, non-syndromic hearing loss | | China | | X. Tang *et al.*, Mitochondrial tRNA(Ser(UCN)) variants in 2651 Han Chinese subjects with hearing loss. *Mitochondrion* **23**, 17-24 (2015). | |
| mtDNA haplogroups and mutations | no association with age-related hearing impairment | | Belgium | | S. Bonneaux *et al*., Inherited mitochondrial variants are not a major cause of age-related hearing impairment in the European population. *Mitochondrion* **11**, 729-734 (2011). | |
| mtDNA 7505C | non-syndromic hearing loss | | China | | X. Tang *et al.*, Maternally inherited hearing loss is associated with the novel mitochondrial tRNA Ser(UCN) 7505T>C mutation in a Han Chinese family. *Mol Genet Metab* **100**, 57-64 (2010). | |
| **Hypertension** |  | |  | |  | |
| mtDNA 4291C | hypertension, hypercho-lesterolemia, hypomagnesemia | | USA | | F. H. Wilson *et al.*, A cluster of metabolic defects caused by mutation in a mitochondrial tRNA. *Science* **306**, 1190-1194 (2004). | |
| mtDNA 4401G | hypertension | | China | | R. Li *et al*., Failures in mitochondrial tRNAMet and tRNAGln metabolism caused by the novel 4401A>G mutation are involved in essential hypertension in a Han Chinese Family. *Hypertension* **54**, 329-337 (2009). | |
| mtDNA 4435G | hypertension | | China | | Z. Lu *et al.*, The tRNAMet 4435A>G mutation in the mitochondrial haplogroup G2a1 is responsible for maternally inherited hypertension in a Chinese pedigree. *Eur J Hum Genet* **19**, 1181-1186 (2011). | |
| mtDNA 4295G | hypertension | | China | | Z. Li, Y. Liu, L. Yang, S. Wang, M. X. Guan, Maternally inherited hypertension is associated with the mitochondrial tRNAIle A4295G mutation in a Chinese family. *Biochem Biophys Res Commun* **367**, 906-911 (2008). | |
| mtDNA haplogroup H | not associated with hypertension | | Russia | | 1. Zhelankin *et al.,* 1A.06: Mitochondrial DNA haplogroup H is associated with subclinical carotid atherosclerosis in Russian population. *J Hypertens* **33** suppl 1:e2 (2015), 10.1097/01.hjh.0000467356.04711.5 | |
| ***mtDNA or Y Chromosome Variant*** | ***Proposed Association (or Lack of Association)*** | | ***Study Location*** | | ***Reference*** | |
| **Hypertension (cont.)** |  | |  | |  | |
| mtDNA haplogroups M, HV, JT, UK | pulmonary arterial hypertension | | USA | | S. Farha *et al*., Mitochondrial Haplogroups and Risk of Pulmonary Arterial Hypertension. *PLoS One* **11**, e0156042 (2016). | |
| mtDNA haplogroup L | reduced risk of pulmonary arterial hypertension | | USA | | S. Farha *et al*., Mitochondrial Haplogroups and Risk of Pulmonary Arterial Hypertension. *PLoS One* **11**, e0156042 (2016). | |
| mtDNA 4263G | hypertension | | China | | S. Wang *et al*., Maternally inherited essential hypertension is  associated with the novel 4263A4G mutation in the mitochondrial tRNAIle gene in a large Han Chinese family. *Circ Res* **108**, 862-870 (2011). | |
| **Infertility** |  | |  | |  | |
| Y chromosome haplogroup K | male infertility | | Latvia | | A. Puzuka *et al.*, Y chromosome—a tool in infertility studies of Latvian population. *Russian Journal of Genetics* **47**, 347-353 (2011). | |
| Y chromosome haplogroups F, K, P, N1 | male infertility | | China | | J. Ran *et al.*, Association study between Y-chromosome haplogroups and susceptibility to spermatogenic impairment in Han People from southwest China. *Genet Mol Res* **12**, 59-66 (2013). | |
| **Bone Disease** |  | |  | |  | |
| mtDNA haplogroup U | increased severity of knee osteoarthritis | | Spain | | I. Rego-Perez, M. Fernandez-Moreno, C. Fernandez-Lopez, J. Arenas, F. Blanco, Mitochondrial DNA haplogroups: role in the prevalence and severity of knee osteoarthritis. *Arthritis Rheum* **58**, 2387-2396 (2008). | |
| mtDNA haplogroup J | reduced risk of knee osteoarthritis | | Spain | | I. Rego-Perez, M. Fernandez-Moreno, C. Fernandez-Lopez, J. Arenas, F. Blanco, Mitochondrial DNA haplogroups: role in the prevalence and severity of knee osteoarthritis. *Arthritis Rheum* **58**, 2387-2396 (2008). | |
| mtDNA haplogroup J | reduced risk of hip osteoarthritis | | Spain | | I. Rego, *et al*., Role of european mitochondrial DNA haplogroups in the prevalence of hip osteoarthritis in Galicia, Northern Spain. *Ann Rheum Dis* **69**, 210-213 (2010). | |
| mtDNA haplogroup X | lower hip bone mineral density (osteoporosis) | | USA | | Y. Guo *et al.*, Mitochondria-wide association study of common variants in osteoporosis. *Ann Hum Genet* **75**, 569-574 (2011). | |
| ***mtDNA or Y Chromosome Variant*** | ***Proposed Association (or Lack of Association)*** | | ***Study Location*** | | ***Reference*** | |
| **Bone Disease (cont.)** | | |  | |  | |
| mtDNA 4823C | lower hip bone mineral density (osteoporosis) | | USA | | Y. Guo *et al.*, Mitochondria-wide association study of common variants in osteoporosis. *Ann Hum Genet* **75**, 569-574 (2011). | |
| mtDNA haplogroup G | knee osteoarthritis | | China | | H. Fang *et al.*, Role of mtDNA haplogroups in the prevalence of knee osteoarthritis in a southern Chinese population. *Int J Mol Sci* **15**, 2646-2659 (2014). | |
| mtDNA haplogroups B, B4 | reduced risk of knee osteoarthritis | | China | | H. Fang *et al.*, Role of mtDNA haplogroups in the prevalence of knee osteoarthritis in a southern Chinese population. *Int J Mol Sci* **15**, 2646-2659 (2014). | |
| mtDNA haplogroups J, T | reduced risk of osteoarthritis | | UK, Spain | | A. Soto-Hermida *et al.*, mtDNA haplogroups and osteoarthritis in different geographic populations. *Mitochondrion* **15**, 18-23 (2014). | |
| mtDNA haplogroups J, TJ | osteoarthritis | | Spain | | J. M. Shen, *et al.,* Role of mtDNA haplogroups in the prevalence of osteoarthritis in different geographic populations: a meta-analysis. *PLoS One* **9**, e108896 (2014). doi:10.1371/journal.pone.0108896 | |
| mtDNA haplogroup TJ | slower osteoarthritis progression | | Spain | | A. Soto-Hermida *et al.*, Mitochondrial DNA haplogroups modulate the radiographic progression of Spanish patients with osteoarthritis. *Rheumatol Int* **35**, 337-344 (2015). | |
| mtDNA haplogroup H | more joint surgery for osteoarthritis | | Spain | | A. Soto-Hermida *et al.,* Mitochondrial DNA haplogroups modulate the radiographic progression of Spanish patients with osteoarthritis. *Rheumatol Int* **35**, 337-344 (2015). | |
| mtDNA haplogroups | no association with osteoarthritis | | UK | | G. Hudson *et al*., No evidence of an association between mitochondrial DNA variants and osteoarthritis in 7393 cases and 5122 controls. *Ann Rheum Dis* **72**, 136-139 (2013). | |
| **AIDS/HIV Antiviral Therapy Complications** | | |  | |  | |
| mtDNA haplogroups U5a, J | accelerated AIDS progression | | USA | | S. L. Hendrickson *et al.*, Mitochondrial DNA haplogroups influence AIDS progression. *AIDS* **22**, 2429-2439 (2008). | |
| mtDNA haplogroups I, W, X, H3 | delayed onset of AIDS | | USA | | S. L. Hendrickson *et al.*, Mitochondrial DNA haplogroups influence AIDS progression. *AIDS* **22**, 2429-2439 (2008). | |
| ***mtDNA or Y Chromosome Variant*** | ***Proposed Association (or Lack of Association)*** | | ***Study Location*** | | ***Reference*** | |
| **AIDS/HIV Antiviral Therapy Complications (cont.)** | | | | |  | |
| mtDNA haplogroup Uk | reduced risk of AIDS | | USA | | S. L. Hendrickson *et al.*, Mitochondrial DNA haplogroups influence AIDS progression. *AIDS* **22**, 2429-2439 (2008). | |
| mtDNA haplogroup L1c | peripheral neuropathy during HIV antiretroviral therapy | | USA | | J. A. Canter *et al.*, African mitochondrial DNA subhaplogroups and peripheral neuropathy during antiretroviral therapy. *J Infect Dis* **201**, 1703-1707 (2010). | |
| mtDNA haplogroup L2 | slower CD4 T-cell recovery during HIV antiretroviral therapy | | USA | | B. J. Grady *et al.*, Mitochondrial genomics and CD4 T-cell count recovery after antiretroviral therapy initiation in AIDS clinical trials group study 384. *J Acquir Immune Defic Syndr* **58**, 363-370 (2011). | |
| mtDNA haplogroup I | lipoatrophy during HIV antiretroviral therapy | | USA | | T. Hulgan *et al.*, European mitochondrial DNA haplogroups and metabolic changes during antiretroviral therapy in AIDS Clinical Trials Group Study A5142. *AIDS* **25**, 37-47 (2011). | |
| mtDNA haplogroup J | reduced risk of neuroretinal disorder during HIV antiretroviral therapy | | USA | | S. L. Hendrickson *et al.*, Genetic variants in nuclear-encoded mitochondrial genes influence AIDS progression. *PLoS One* **5**, e12862 (2010), 10.1371/journal.pone.0012862. | |
| mtDNA haplogroup L2 | slower CD4 T-cell recovery during HIV antiretroviral therapy | | USA | | B. Aissani *et al.*, Mitochondrial DNA variation and virologic and immunological HIV outcomes in African Americans. *AIDS* **28**, 1871-1878 (2014). | |
| mtDNA haplogroups J, T | slower CD4 T-cell recovery during HIV antiretroviral therapy | | Spain | | M. Guzman-Fulgencio *et al.*, European mitochondrial haplogroups are associated with CD4+ T cell recovery in HIV-infected patients on combination antiretroviral therapy. *J Antimicrob Chemother* **68**, 2349-2357 (2013). | |
| European mtDNA haplogroups | not associated with hepatitis C virus treatment response | | Spain | | M. Guzmán-Fulgencio *et al*., European mitochondrial haplogroups are not associated with hepatitis C virus (HCV) treatment response in HIV/HCV-coinfected patients. *HIV Med* **15**, 425-430 (2014). | |
| mtDNA haplogroup A | susceptibility to AIDS | | China | | H. W. Wang, *et al.,* Mitochondrial DNA Haplogroup A may confer a genetic susceptibility to AIDS group from Southwest China.*Mitochondrial DNA A DNA Mapp Seq Anal* **27**, 2221-2224 (2016). | |
| ***mtDNA or Y Chromosome Variant*** | ***Proposed Association (or Lack of Association)*** | | ***Study Location*** | | ***Reference*** | |
| **AIDS/HIV Antiviral Therapy Complications (cont.)** | | | | |  | |
| mtDNA haplogroup B | less neurocognitive impairment during HIV antiretroviral therapy | | USA | | T. Hulgan *et al.,* Mitochondrial DNA Haplogroups and Neurocognitive Impairment During HIV Infection. *Clin Infect Dis* **61**, 1476-1484 (2015). | |
| mtDNA haplogroup H | faster CD4 T-cell recovery during HIV antiretroviral therapy | | Spain | | M. Guzman-Fulgencio *et al.*, European mitochondrial haplogroups are associated with CD4+ T cell recovery in HIV-infected patients on combination antiretroviral therapy. *J Antimicrob Chemother* **68**, 2349-2357 (2013). | |
| **Neurodegenerative Conditions/Treatment Response** | | | | |  | |
| mtDNA haplogroup H (7028C) | earlier onset of Huntington's disease | | Germany | | L. Arning *et al.*, Mitochondrial haplogroup H correlates with ATP levels and age at onset in Huntington disease. *J Mol Med (Berl)* **88**, 431-436 (2010). | |
| mtDNA haplogroup H (7028C) | late onset Alzheimer's disease | | Spain | | E. Coto *et al.*, Late-onset Alzheimer's disease is associated with mitochondrial DNA 7028C/haplogroup H and D310 poly-C tract heteroplasmy. *Neurogenetics* **12**, 345-346 (2011). | |
| mtDNA haplogroups J1c, J2, U4, U5a1, K | reduced risk of Parkinson's disease | | Poland | | K. Gaweda-Walerych *et al.*, Mitochondrial transcription factor A variants and the risk of Parkinson's disease. *Neurosci Lett* **469**, 24-29 (2010). | |
| mtDNA haplogroup H | Alzheimer's and Parkinson's diseases | | Spain | | A. Gomez-Duran *et al.*, Unmasking the causes of multifactorial disorders: OXPHOS differences between mitochondrial haplogroups. *Hum Mol Genet* **19**, 3343-3353 (2010). | |
| mtDNA haplogroup Uk | reduced risk of Alzheimer's and Parkinson's disease | | Spain | | A. Gomez-Duran *et al.*, Unmasking the causes of multifactorial disorders: OXPHOS differences between mitochondrial haplogroups. *Hum Mol Genet* **19**, 3343-3353 (2010). | |
| mtDNA haplogroup Uk | Alzheimer's disease | | USA, Canada | | A. Lakatos *et al.*, Association between mitochondrial DNA variations and Alzheimer's disease in the ADNI cohort. *Neurobiol Aging* **31**, 1355-1363 (2010). | |
| ***mtDNA or Y Chromosome Variant*** | ***Proposed Association (or Lack of Association)*** | | ***Study Location*** | | ***Reference*** | |
| **Neurodegenerative Conditions/Treatment Response (cont.)** | | | | |  | |
| mtDNA haplogroups | no association with Alzheimer's disease | | N.A. | | M. Mancuso, V. Calsolaro, D. Orsucci, G. Siciliano, L. Murri, Is there a primary role of the mitochondrial genome in Alzheimer's disease? *J Bioenerg Biomembr* **41**, 411-416 (2009). | |
| mtDNA haplogroup E1 | Parkinson's disease | | Guam | | D. M. Reiff *et al.*, Inherited and somatic mitochondrial DNA mutations in Guam amyotrophic lateral sclerosis and parkinsonism-dementia. *Neurol Sci* **32**, 883-892 (2011). | |
| mtDNA haplogroup E2 | reduced risk of Parkinson's disease | | Guam | | D. M. Reiff *et al.*, Inherited and somatic mitochondrial DNA mutations in Guam amyotrophic lateral sclerosis and parkinsonism-dementia. *Neurol Sci* **32**, 883-892 (2011). | |
| mtDNA haplogroup H5 | Alzheimer's disease | | Italy | | A. Santoro *et al.*, Evidence for sub-haplogroup h5 of mitochondrial DNA as a risk factor for late onset Alzheimer's disease. *PLoS One* **5**, e12037 (2010), 10.1371/journal.pone.0012037. | |
| mtDNA haplogroups | no association with Parkinson's disease | | USA | | D. K. Simon *et al.*, Maternal inheritance and mitochondrial DNA variants in familial Parkinson's disease. *BMC Med Genet* **11**, 53 (2010), 10.1186/1471-2350-11-53. | |
| mtDNA haplogroup H | Parkinson's disease | | UK | | G. Hudson *et al*., Two-stage association study and meta-analysis of mitochondrial DNA variants in Parkinson disease. *Neurology* **80**, 2042-2048 (2013). | |
| mtDNA haplogroups J, K, T | reduced risk of Parkinson's disease | | UK | | G. Hudson *et al*., Two-stage association study and meta-analysis of mitochondrial DNA variants in Parkinson disease. *Neurology* **80**, 2042-2048 (2013). | |
| mtDNA haplogroup D | Parkinson's disease | | China | | Y. F. Chen *et al*., Mitochondrial DNA Haplogroups and the Risk of Sporadic Parkinson's Disease in Han Chinese. *Chin Med J (Engl)* **128**, 1748-1754 (2015). | |
| mtDNA haplogroup B | reduced risk of Parkinson's disease | | China | | Y. F. Chen *et al*., Mitochondrial DNA Haplogroups and the Risk of Sporadic Parkinson's Disease in Han Chinese. *Chin Med J (Engl)* **128**, 1748-1754 (2015). | |
| ***mtDNA or Y Chromosome Variant*** | ***Proposed Association (or Lack of Association)*** | | ***Study Location*** | | ***Reference*** | |
| **Neurodegenerative Conditions/Treatment Response (cont.)** | | | | |  | |
| mtDNA haplogroup B5 | Alzheimer's disease | | China | | R. Bi *et al*., Mitochondrial DNA haplogroup B5 confers genetic susceptibility to Alzheimer's disease in Han Chinese. *Neurobiol Aging* **36**, 1604.e7-16 (2015). | |
| mtDNA haplogroup L1 | Alzheimer's disease | | USA | | G. J. Tranah *et al.*, Mitochondrial DNA sequence associations with dementia and amyloid-beta in elderly African Americans. *Neurobiol Aging* **35**, 442 e441-448 (2014). | |
| **Opthalmic Diseases/Conditions** | | |  | |  | |
| mtDNA 10609C, 10663C | Leber's hereditary optic neuropathy | | Kuwait | | R. Behbehani *et al*., ND4L gene concurrent 10609T > C and 10663T > C mutations are associated with Leber's hereditary optic neuropathy in a large pedigree from Kuwait. *Brit J Ophthalmol* **98**, 826-831 (2014). | |
| mtDNA haplogroups H, R | keratoconus | | Saudi Arabia | | K.K. Abu-Amero *et al*., Association of mitochondrial haplogroups H and R with keratoconus in Saudi Arabian patients. *Invest Ophth Vis Sci* **55**, 2827-2831 (2014). | |
| mtDNA haplogroup T | increased risk of diabetic retinopathy | | Austria | | B. Kofler et al., Mitochondrial DNA haplogroup T is associated with coronary artery disease and diabetic retinopathy: a case control study. *BMC Med Genet* **10**: 35 (2009), 10.1186/1471-2350-10-35. | |
| mtDNA 14484C | Leber's hereditary optic neuropathy | | Various | | T. M. Bosley, K. K. Abu-Amero, Assessing mitochondrial DNA nucleotide changes in spontaneous optic neuropathies. *Ophthalmic Genet* **31**, 163-172 (2010); N.A. Khan *et al*., Haplogroup heterogeneity of LHON patients carrying the m.14484T > C mutation in India. *Invest Ophth Vis Sci* **54**, 3999-4005 (2013). | |
| mtDNA haplogroup T2 | age-related macular degeneration | | USA, Australia | | J. P. SanGiovanni *et al.*, Mitochondrial DNA variants of respiratory complex I that uniquely characterize haplogroup T2 are associated with increased risk of age-related macular degeneration. *PLoS One* **4**, e5508 (2009), 10.1371/journal.pone.0005508. | |
| mtDNA haplogroup L | primary open-angle glaucoma | | Saudi Arabia | | K. K. Abu-Amero *et al*., Mitochondrial DNA lineages of African origin confer susceptibility to primary open-angle glaucoma in Saudi patients. *Mol Vis* **17**, 1468-1472 (2011). | |
| ***mtDNA or Y Chromosome Variant*** | ***Proposed Association (or Lack of Association)*** | | ***Study Location*** | | ***Reference*** | |
| **Opthalmic Diseases/Conditions (cont.)** | | |  | |  | |
| mtDNA haplogroup N1 | reduced risk of primary open-angle glaucoma | | Saudi Arabia | | K. K. Abu-Amero *et al*., Mitochondrial DNA lineages of African origin confer susceptibility to primary open-angle glaucoma in Saudi patients. *Mol Vis* **17**, 1468-1472 (2011). | |
| mtDNA haplogroups L2, T | pseudoexfoliation glaucoma | | Saudi Arabia | | K. K. Abu-Amero *et al*., Eurasian and Sub-Saharan African mitochondrial DNA haplogroup influences pseudoexfoliation glaucoma development in Saudi patients. *Mol Vis* **17**, 543-547 (2011). | |
| mtDNA haplogroup N1 | reduced risk of pseudoexfoliation glaucoma | | Saudi Arabia | | K. K. Abu-Amero *et al*., Eurasian and Sub-Saharan African mitochondrial DNA haplogroup influences pseudoexfoliation glaucoma development in Saudi patients. *Mol Vis* **17**, 543-547 (2011). | |
| mtDNA 11778A | Leber's hereditary optic neuropathy | | Various | | T. M. Bosley, K. K. Abu-Amero, Assessing mitochondrial DNA nucleotide changes in spontaneous optic neuropathies. *Ophthalmic Genet* **31**, 163-172 (2010). | |
| mtDNA 3460A | Leber's hereditary optic neuropathy | | Various | | T. M. Bosley, K. K. Abu-Amero, Assessing mitochondrial DNA nucleotide changes in spontaneous optic neuropathies. *Ophthalmic Genet* **31**, 163-172 (2010). | |
| mtDNA haplogroup B5a1 | Leber's hereditary optic neuropathy | | Thailand | | S. Kaewsutthi *et al*., Mitochondrial haplogroup background may influence Southeast Asian G11778A Leber hereditary optic neuropathy. *Invest Ophth Vis Sci* **52**, 4742-4748 (2011). | |
| mtDNA haplogroup F | reduced risk of Leber's hereditary optic neuropathy | | Thailand | | S. Kaewsutthi *et al*., Mitochondrial haplogroup background may influence Southeast Asian G11778A Leber hereditary optic neuropathy. *Invest Ophth Vis Sci* **52**, 4742-4748 (2011). | |
| mtDNA haplogroup U | reduced risk of exfoliation glaucoma | | Germany | | C. Wolf *et al.*, Mitochondrial haplogroup U is associated with a reduced risk to develop exfoliation glaucoma in the German population. *BMC Genet* **11**, 8 (2010), 10.1186/1471-2156-11-8. | |
| mtDNA haplogroups A2, C | later onset/reduced impact of Leber's hereditary optic neuropathy | | Chile | | P. Romero *et al.,* Pan-American mDNA haplogroups in Chilean patients with Leber's hereditary optic neuropathy. *Mol Vis* **14,** 334-40 (2014). | |
| ***mtDNA or Y Chromosome Variant*** | ***Proposed Association (or Lack of Association)*** | | ***Study Location*** | | ***Reference*** | |
| **Opthalmic Diseases/Conditions (cont.)** | | |  | |  | |
| mtDNA 14502C, 11778A | Leber's hereditary optic neuropathy | | China | | P. Jiang *et al.,* Biochemical evidence for a mitochondrial genetic modifier in the phenotypic manifestation of Leber's hereditary optic neuropathy-associated mitochondrial DNA mutation. *Hum Mol Genet* **Jul 17**. pii: ddw199 (2016). | |
| mtDNA 3635A, 14502C | Leber's hereditary optic neuropathy | | China | | X. Jin *et al.*, Leber's Hereditary Optic Neuropathy is Associated with Compound Primary Mutations of Mitochondrial ND1 m.3635G > A and ND6 m.14502 T > C. *Ophthalmic Genet* **36**, 291-298 (2015). | |
| mtDNA 16111, 16362, 16319, 1736, 12007 | age-related macular degeneration | | USA | | N. A. Resrepo, *et al.*, Mitochondrial variation and the risk of age-related macular degeneration across diverse populations. *Pac Symp Biocomput.* 243-54 (2015). | |
| mtDNA haplogroups J, T, U | age-related macular degeneration | | USA | | M. C. Kenney *et al*., Mitochondrial DNA haplogroups confer differences in risk for age-related macular degeneration: a case control study. *BMC Med Genet* **14** (2014), 10.1186/1471-2350-14-4. | |
